# Determination of Tobramycin in M_9_ Medium by LC-MS/MS: Signal Enhancement by Trichloroacetic Acid

**DOI:** 10.1155/2018/7965124

**Published:** 2018-04-26

**Authors:** Liusheng Huang, Janus Anders Juul Haagensen, Davide Verotta, Vincent Cheah, Alfred M. Spormann, Francesca Aweeka, Katherine Yang

**Affiliations:** ^1^Department of Clinical Pharmacy, School of Pharmacy, University of California San Francisco, San Francisco, CA, USA; ^2^Novo Nordisk Foundation Center for Biosustainability, Technical University of Denmark, 2800 Kongens Lyngby, Denmark; ^3^Department of Civil and Environmental Engineering, Stanford University, Palo Alto, CA, USA

## Abstract

It is well known that ion-pairing reagents cause ion suppression in LC-MS/MS methods. Here, we report that trichloroacetic acid increases the MS signal of tobramycin. To support studies of an *in vitro* pharmacokinetic/pharmacodynamic simulator for bacterial biofilms, an LC-MS/MS method for determination of tobramycin in M_9_ media was developed. Aliquots of 25 *μ*L M_9_ media samples were mixed with the internal standard (IS) tobramycin-d_5_ (5 *µ*g/mL, 25 *µ*L) and 200 *µ*L 2.5% trichloroacetic acid. The mixture (5 *µ*L) was directly injected onto a PFP column (2.0 × 50 mm, 3 *µ*m) eluted with water containing 20 mM ammonium formate and 0.14% trifluoroacetic acid and acetonitrile containing 0.1% trifluoroacetic acid in a gradient mode. ESI^+^ and MRM with ion *m*/*z* 468 → 324 for tobramycin and *m*/*z* 473 → 327 for the IS were used for quantification. The calibration curve concentration range was 50–25000 ng/mL. Matrix effect from M_9_ media was not significant when compared with injection solvents, but signal enhancement by trichloroacetic acid was significant (∼3 fold). The method is simple, fast, and reliable. Using the method, the *in vitro* PK/PD model was tested with one bolus dose of tobramycin.

## 1. Introduction

**Figure 1:**
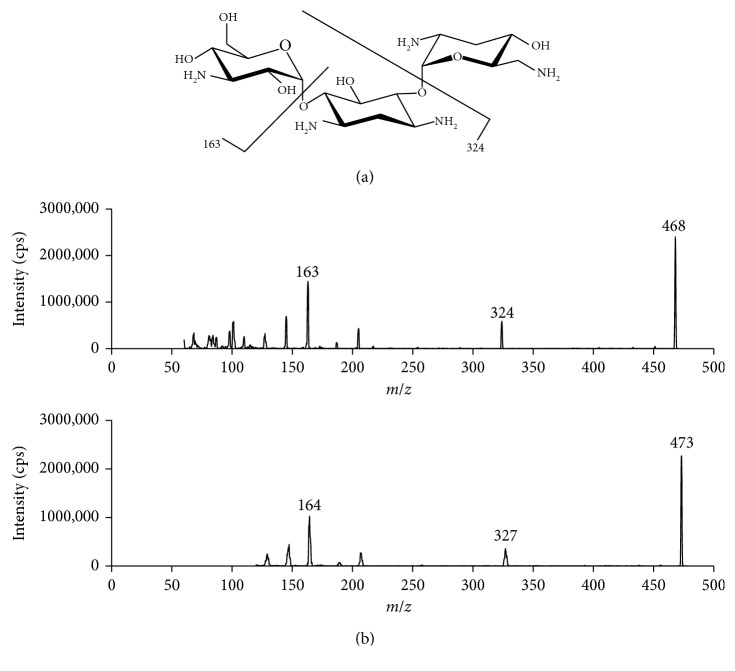
Product ion spectra of tobramycin (a) and deuterated tobramycin (b).

Tobramycin (TBM) is an aminoglycoside antibiotic widely used for the treatment of multidrug-resistant Gram-negative bacterial infections by inhibiting protein synthesis and altering integrity of the bacterial cell membrane [1]. It is also named 3′-deoxykanamycin B, nebramycin 6, and chemically O-3-amino-3-deoxy-*α*-D-glucopyranosyl-(1-6)-O-[2,6-diamino-2,3,6-trideoxy-*α*-D-ribo-hexopyranisyl-(1-4)]-2-deoxy-D-streptamine (Figure 1). It is water soluble and stable at ambient temperature at a wide range of pH 1–11 [2].

To support pharmacokinetic (PK) and pharmacodynamic (PD) studies of TBM for biofilm-mediated infections using an *in vitro* model, an analytical method to quantitate TBM in M_9_ medium is needed. Two considerable challenges in determination of TBM in biological matrices are (1) poor retention on commonly used reverse-phase HPLC columns due to its higher hydrophilicity and (2) lack of chromophores for detection. Numerous assays have been reported including HPLC coupled with UV [3], electrochemical [4, 5], or fluorescence detectors [6], and these assays lack sensitivity and usually require derivatization. LC-MS/MS assays have also been reported, but the sensitivity of these assays requires concentrations ≥100 ng/mL [7–9]. Trichloroacetic acid (TCA) has been used in sample preparation to remove proteins, especially for hydrophilic analytes, with the advantage of direct injection of resulting sample solution [10]. We found that TCA not only increased the retention time but also the MS signal of TBM. Built on this observation, we report a simple LC-MS/MS method to determine TBM in M_9_ medium using TCA as the ion pair reagent in the injection sample instead of the mobile phase. In addition, this assay utilized a PFP column, which yielded a better retention factor for TBM (*k* = 1.8). The calibration range was 50–25000 ng/mL.

## 2. Experimental

### 2.1. Chemicals and Reagents

Tobramycin was purchased from Sigma-Aldrich (St. Louis, MO, USA). Deuterated tobramycin (TBM-d_5_) was purchased from Toronto Research Chemicals (North York, Ontario, Canada). Formulated tobramycin (20 mg/2 mL, for IM or IV use) was obtained from APP Pharmaceuticals, LLC (Schaumburg, IL, USA). Common solvents (HPLC grade) and reagents (Certified ACS) were obtained from Thermo-Fisher Sci. (Fair Lawn, NJ, USA). M_9_ minimal salts ×5 solution was prepared by dissolving 2.82 g Difco™ M_9_ minimal salts (BD, Sparks, MD, USA) in 50 mL water. M_9_ medium was prepared by adding 10 mL M_9_ minimal salts ×5 solution, 5 *µ*L 1 M CaCl_2_, 50 *µ*L 1 M MgSO_4_, and 13.5 *µ*L 20% glucose to 40 mL water.

### 2.2. Instrumental

The LC-MS/MS system consists of an AB Sciex API5000 Tandem Mass Spectrometer, two Shimadzu Prominence 20AD^XR^ UFLC pumps, and an SIL-20AC^XR^ autosampler managed with Analyst® 1.6.2 (AB Sciex, Redwood City, CA, USA). The gases for the MS system were supplied by an LC-MS gas generator (Source 5000™, Parker Balston Inc., Haverhill, MA, USA). LC conditions were as follows: separation was achieved on a Pursuit PFP column (2.1 × 50 mm, 3 *µ*m) (Agilent Tech. Inc., Santa Clara, CA, USA). Mobile phase A was 20 mM NH_4_FA 0.14% trifluoroacetic acid (TFA) and B was 0.1% TFA in acetonitrile (MeCN). Five-microliter sample was injected onto the column eluted at a flow rate of 0.4 mL/min in a gradient program consisting of 5% solvent B (0–0.10 min), from 5 to 20% B (0.10–1.50 min), from 20 to 80% B (1.50–1.51 min), 80% B (1.51–2.00 min), 80%–5% B (2.00–2.01 min), and 5% B (2.01–3.00 min). Retention times for TBM and the internal standard (IS) were both 0.84 min. The divert valve was set to direct the LC eluent to the mass spectrometer (MS) source at 0.6 min and to the waste line at 2.9 min. The MS conditions for TBM and the IS were optimized by separate infusion of 200 ng/mL TBM and 400 ng/mL deuterated TBM in 0.1% formic acid into the MS at a flow rate of 15 and 25 *µ*L/min constantly while adjusting MS parameters with autotune followed by manual adjustment to achieve the maximal signal. The ions *m*/*z* 468 → 324 for TBM and *m*/*z* 473 → 327 for the IS were used for quantification in the multiple reaction monitoring (MRM) mode. The optimized compound-dependent MS parameters were 121 V (DP), 21 V (CE), and 26 V (CXP) for both TBM and the IS. DP was declustering potential, CE was collision energy, and CXP was collision cell exit potential. The instrument-dependent parameters were optimized by flow injection analysis (FIA): an aliquot of 5 *µ*L 200 ng/mL TBM was repeatedly injected into the LC-MS/MS system while LC flow was maintained at 0.4 mL/min 50% B isocratically without column in the line. The optimized MS parameters were as follows: MS source was the TurboIon Spray ionization in positive mode (ESI^+^) with turbo heater set at 500°C, curtain gas was nitrogen at 40 psi, nebulizer gas (gas 1) and auxiliary (Turbo) gas (gas 2) were zero air set at 50 psi and 60 psi, respectively, collision-deactivated association gas was nitrogen at 12 psi, and ionspray voltage was 5500 V. Data were processed with Analyst 1.6.2. (AB Sciex, Redwood City, CA, USA).

### 2.3. Preparation of Calibrators, Quality Controls, and Internal Standard

As TBM used in the *in vitro* biofilm PK/PD model contains formulation ingredients, calibrators and quality controls (QCs) were prepared from formulated TBM (20 mg/2 mL) with serial dilution in M_9_ medium to match the matrix in unknown samples. Calibrators consists of 50, 100, 250, 500, 1000, 2500, 5000, 10000, and 25000 ng/mL. QCs consist of 150, 1500, 20000, and 40000 ng/mL, designated as low-, medium-, high-, and extrahigh QC. The internal standard TBM-d_5_ solution was prepared in water by serial dilution at a final concentration of 5000 ng/mL. The IS solution needs to stand on bench overnight before use.

### 2.4. Sample Preparation

M_9_ samples (25 *µ*L) were pipetted into 1 mL glass autosampler vials, to which were added 25 *µ*L IS (5 *µ*g/mL TBM-d_5_) and 200 *µ*L 2.5% TCA. After vortex mixed, the samples were placed in the autosampler tray. If the samples were collected from M_9_ medium flowing through bacterial biofilm, the samples were centrifuged at 20000*g* for 3 min before adding to the sample vial. Injection volume was 5 *µ*L.

### 2.5. Validation

The method was validated in terms of precision, accuracy, matrix effect, and stability, following the procedures as described previously [10]. One set of calibrators was processed for each run and injected in the beginning of the batch run. Calibration curves were constructed by linear regression of the peak area ratio of the analyte to the IS (*y*-axis) versus the nominal analyte concentrations (*x*-axis) with a weighting factor of 1/*x*. The lower limit of quantification (LLOQ) was established with precision and accuracy <20%. Intraday precision and accuracy were determined by analysis of at least five replicates of each QC sample at low (150 ng/mL), medium (1500 ng/mL), and high (20000 ng/mL) concentration levels extracted with a set of calibrators in one batch. The same procedure was repeated on at least 2 different days with new samples to determine interday precision and accuracy (total: *n* ≥ 15 per concentration level). Precision was reported as relative standard deviation (RSD) and accuracy as percent deviation from the nominal concentration (% dev.). Matrix effect was evaluated as follows: TBM was spiked at the concentrations of 300, 1500, and 20000 ng/mL in water and M_9_ medium, respectively. Three aliquots of each sample were processed as described above (Section 2.4). The peak areas and peak area ratios of TBM in M_9_ medium were compared to those in water. Values within 100 ± 15% were considered as no significant matrix effect from M_9_ medium. To evaluate partial volume accuracy, 12.5 *µ*L extrahigh QC at 40000 ng/mL was mixed with 12.5 *µ*L M_9_ medium and processed as described in Section 2.4. Stability was evaluated in the following conditions: room temperature (21–25°C) for 5 days, 3 days on the autosampler rack, 3 freeze-thaw cycles, and 6 days at −70°C. Stability of freshly prepared IS working solution was evaluated at room temperature for 24 hr and 5 days. Effects of concomitant drugs (e.g., meropenem and colistin) on quantification were evaluated by spiking them in the QC samples at a final concentration of 110 *µ*g/mL meropenem (MP) and 20 *µ*g/mL colistin. The measured concentrations of TBM were compared to the QC samples without these concomitant drugs.

### 2.6. Application

This method was used to validate a novel dynamic PK/PD model designed to study the effects of human-simulated antibiotic concentrations on *Pseudomonas aeruginosa* biofilms grown *in vitro* [11]. TBM, in conjunction with a *β*-lactam antibiotic such as MP, is recommended for the treatment of multidrug-resistant *Pseudomonas aeruginosa* lung infection in patients with cystic fibrosis [12]. While the formation of bacterial biofilms in the lung is a characteristic of chronic lung infection in patients with cystic fibrosis, the PD of antibiotics on biofilms is largely unknown. The concentration-time curves of single and multiple intravenous bolus doses of TBM were simulated based on human population PK parameters [13]. The target TBM peak concentration, based on a dose of 10 mg/kg in a 70 kg adult, was 32.79 mg/L with an associated *t*_1/2_ = 2.75 h. Samples were taken at *t* = 0, 1, 2, 4, 6, 8, 16, and 24 hr from the main feeding bottle and the tubing outlets from three flow cells with bacterial biofilm. All samples were shipped to our analytical lab on the same day with dry ice overnight delivery and stored at −70°C freezer until analysis. Samples were typically analyzed within a week.

## 3. Results and Discussion

### 3.1. LC-MS/MS Optimization

TBM contains five amine groups (Figure 1), making electrospray ionization in positive mode (ESI^+^) the choice of the ion source. The ion *m*/*z* 468 → 324 was chosen for quantification for its signal abundancy and selectivity. Compared to product ion *m*/*z* 163, *m*/*z* 324 has less background signal. The deuterated TBM was used as the IS. However, the deuteration positions were not identified. MS scan showed that multiple forms of deuterated TBM exist, with the most abundant protonated molecule at *m*/*z* 473. Therefore, ion *m*/*z* 473 → 327 was chosen for the IS. The signal of the ion *m*/*z* 473 → 327 decreased gradually in the first few hours but remained stable after the IS solution stood on bench overnight. These observations suggested that deuteration most likely occurred on amine groups, and the stable form of IS contains a deuterium atom on each amine group (Figure 1).

**Figure 2:**
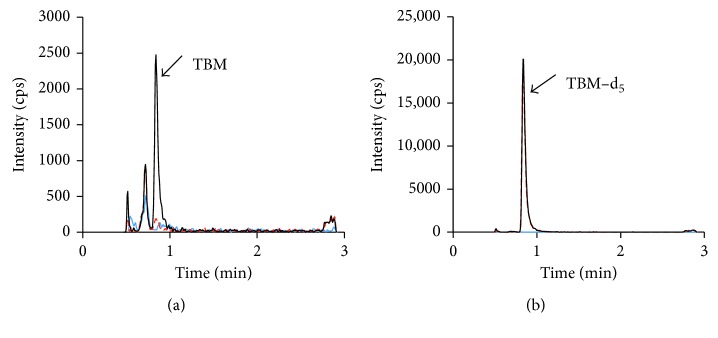
Chromatograms of blank M_9_ medium (blue solid line), blank M_9_ medium spiked with IS (red dash line), and TBM at LLOQ level (black solid line). (a) TBM channel; (b) the IS channel.

Having 5 amine groups and 5 hydroxyl groups also makes TBM hardly retain on reverse-phase LC columns. Ion pair reagent TFA and TCA in the mobile phase could help to retain polar amino molecules on the reverse-phase columns; however, sensitivity may be compromised due to ion suppression. Previously, we found that TFA could change retention time of isoniazid when added into sample before injection (Supplementary Material Figure S1). However, TFA did not improve the TBM peak. Cheng et al. used TCA to modify retention time of aminoglycoside compounds [14]. We found that when the sample contained 2% TCA with a 5 *µ*L injection volume, longer retention time of TBM was observed (Supplementary Material Figure S2). Under the final LC condition, the TBM peak was sufficiently separated from the matrix-generated peaks (Figure 2). The retention time *t*_R_ = 0.839 min, the estimated dead volume is 0.68*πr* ^2^ *L* = 0.118 mL, and retention factor *k* = 1.84.

**Figure 3:**
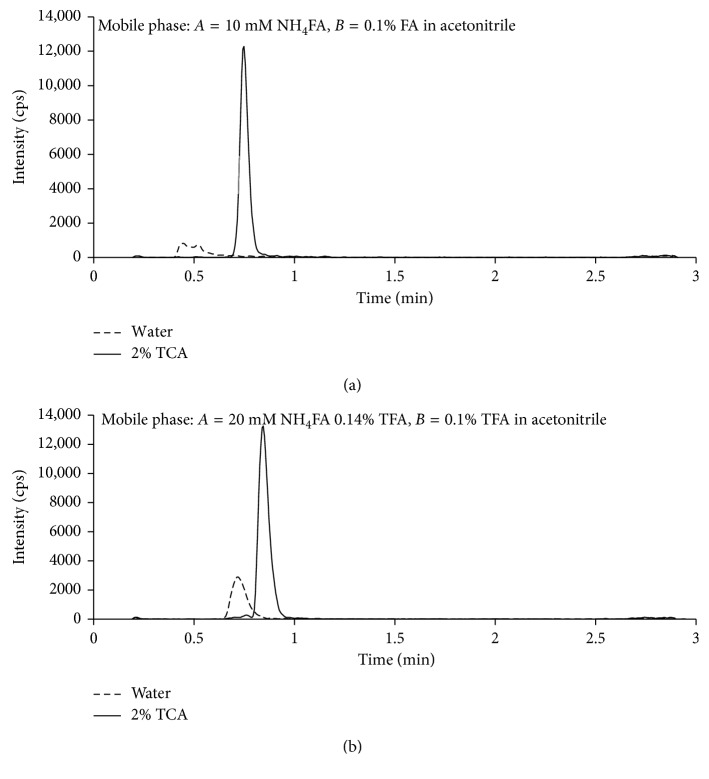
Impact of mobile phase solvents and sample solvents on peak shape, retention time, and signal intensity of TBM. Sample solvents: water (dash line) and 2% TCA (solid line). Mobile phase solvents: 10 mM NH_4_FA (pH 4.0)-0.1% FA in MeCN (a) and 20 mM NH_4_FA 0.14% TFA-0.1% TFA in MeCN (b).

Unexpectedly, TCA also enhanced MS response of TBM. Two different sample solvents (water and 2% TCA) and two sets of mobile phase solvents were tested: (1) *A* = 10 mM NH_4_FA at pH 4.0; *B* = 0.1% FA in MeCN and (2) *A* = 20 mM NH_4_FA 1.4% TFA;, *B* = 0.1% TFA in MeCN, using the same gradient elution method. With the commonly used mobile phase solvents (set 1), the peak shape for TBM was poor if injection solvent is water, while 2% TCA in the sample improved peak shape, signal intensity, and retention time significantly (Figure 3(a)); with mobile phase solvent set 2, the signal intensity and retention time of TBM improved further (Figure 3(b)). This improvement is critical as the interference peak from M_9_ medium was then separated from the TBM peak (Figure 2). The exact mechanism of signal enhancement by TCA is unknown. Cheng et al. thought that reduced matrix effect with longer retention time contributed to the signal enhancement [14], but we observed signal enhancement in neat solution (Figure 3). The possible reason could be that TCA limited multiple charges of TBM and thus increased monocharged molecular ion ([M+H]^+^). In addition, we observed that MS response of the IS (TBM-d_5_) was also increased with the increase of TBM concentration, suggesting ion enhancement of coeluting compounds. This should not affect quantification as IS was added to all samples, and the TBM signal increased accordingly. This was confirmed with the excellent linearity of calibration curve.

### 3.2. Validation

Based on our initial simulation, the TBM trough concentration is expected to be >250 ng/mL. Therefore, the LLOQ in this assay was initially set at 250 ng/mL, the upper limit of quantification was set at 25000 ng/mL, and validation was performed with low (300 ng/mL), medium (1500 ng/mL), and high (20000 ng/mL) QCs. After tested the *in vitro* biofilm model, we found that the trough TBM concentration fell below 250 ng/mL, and thus, we lowered the LLOQ to 50 ng/mL and the low QC level to 150 ng/mL accordingly. Validation of intraday/interday precision and accuracy and interference of concomitant drugs were repeated with the new low QC concentration.

#### 3.2.1. Calibration Range

At the LLOQ concentration (50 ng/mL), the signal intensity was 2100–2400 cps (peak area, 6600–7900) and signal-to-noise ratio *S*/*N* = 30–48 (Figure 2). This LLOQ is lower than others reported in literature. A recent study reported an LLOQ at 100 ng/mL. The detector was the same as ours, but heptafluorobutyric acid was used as ion pair reagent in the mobile phase and sample reconstitution [9]. The calibration curve was constructed with least square linear regression weighted by 1/*x*. The interday backcalculated concentrations of calibrators over 3 days are listed in Table 1. The precision is within 10% and accuracy (percent deviation from the nominal value) is within ±10%, too. Representative MRM ion chromatograms of TBM from M_9_ medium (double blank), M_9_ medium spiked with IS (blank), and LLOQ samples are shown in Figure 2.

#### 3.2.2. Precision and Accuracy

The intraday precision (*n* = 6) was within 7% at low, medium, and high concentrations. The interday precision, calculated with the individual mean concentration from 3 days, was within 5% at the three concentration levels. The intra- and interday accuracy was all within 15%. At the LLOQ levels, the precision and accuracy met the criteria of <20% (Table 2).

#### 3.2.3. Matrix Effect

The matrix effect of M_9_ medium on both TBM and IS signals is within 100 ± 15% (Table 3), suggesting that the matrix effect of M_9_ medium was not significant. The matrix effect on the peak area ratio was even smaller, suggesting that IS compensated the matrix effect.

#### 3.2.4. Partial Sample Volume Accuracy

As the target Cmax is 40000 ng/mL, we evaluated accuracy of the assay with an extrahigh QC (40000 ng/mL) when half sample volume was used. The precision and accuracy from six replicates of analysis were 2.2% and 2.6%, respectively. Therefore, samples above the upper limit of quantification could be analyzed with a partial volume.

#### 3.2.5. Stability

TBM was stable in M_9_ medium. No significant degradation was found under tested condition (Table 4). Further investigation is ongoing to define long-term stability in −70°C freezer.

#### 3.2.6. Evaluation of Concomitant Drug Interference

The samples from the supported study are expected to contain MP and colistin; therefore, impact of these drugs on quantification of TBM was evaluated. In the presence of 110 *µ*g/mL MP and 20 *µ*g/mL colistin, the low, medium, and high QC samples could still be quantified accurately, with a small percent deviation from the samples without these drugs (Table 5).

### 3.3. Application

**Figure 4:**
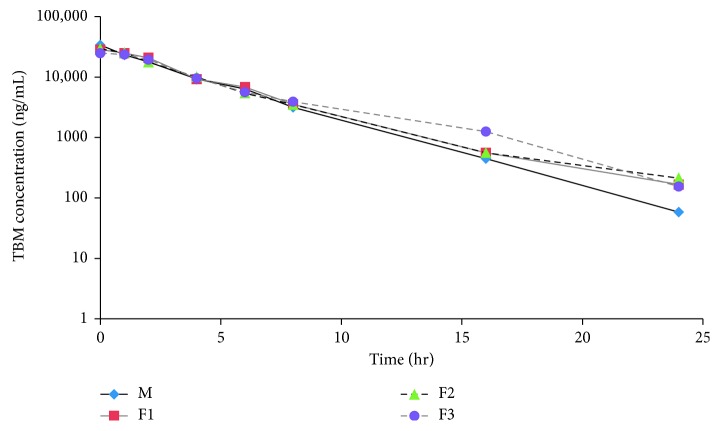
Concentration-time profile of tobramycin from an *in vitro* PK/PD biofilm model. Samples were taken from the feeding bottle (M) and the tubing outlets from three flow cells with bacterial biofilm (F1, F2, and F3) at designated time intervals.

The method was applied to determine TBM concentrations used in an *in vitro* PK/PD biofilm simulator. The PK/PD analysis was reported elsewhere [15]. A representative concentration-time curve from the model is showed in Figure 4. The results demonstrate that the sensitivity of the method met the requirement of the intended study.

## 4. Conclusion

TCA not only improves peak shape and retention time of TBM but also increases MS signal intensity of TBM. Using a simple dilution with ion pairing reagent TCA, a sensitive LC-MS/MS method was developed and validated for determination of TBM in bacterial M_9_ medium. The LLOQ was 50 ng/mL. The sensitivity of the assay met the requirement of the intended PK/PD study in an *in vitro* biofilm model system.

TCA has been used to increase retention time and sensitivity for quantification of gentamicin, kanamycin, and apramycin [14]. Here, we demonstrated application of TCA to quantification of TBM. We speculate this approach could be generalized: by addition of ion-pairing agents to samples instead of adding to mobile phase solvents, we could extend the retention time of analytes and even increase sensitivity. Acidic ion-pairing agents such as TFA and TCA could be applied to basic polar analytes such as amine-containing analytes, and basic ion-pairing agents could be added to samples of acidic polar analytes. Nevertheless, the concentration of the ion-pairing agent is critical, and selection of the ion-pairing agent is also critical.

## Figures and Tables

**Table 1:** Interday average backcalculated standard concentrations (*n* = 3).

Nominal concentration (ng/mL)	50	100	250	500	1000	2500	5000	10000	25000	*R*
Mean (ng/mL)	50.1	91.6	236	521	1043	2663	4973	9977	24800	0.9992
Precision (RSD, %)	3.82	6.11	3.31	2.98	3.37	2.50	8.06	3.72	2.91	0.0379
Accuracy (% dev.)	0.13	−8.37	−5.60	4.13	4.33	6.53	−0.53	−0.23	−0.80	
*n*	3	3	3	3	3	3	3	3	3	

**Table 2:** Intra- and interday precision and accuracy.

	Intraday				Interday			
Nominal (ng/mL)	50.0	150	1500	20000	50.0	150	1500	20000
Mean (ng/mL)	43.0 to 50.9	150 to 159	1533 to 1653	20450 to 20650	46.8	153	1591	20572
Precision (RSD) (%)	3.0 to 16.9	4.4 to 6.7	2.1 to 3.5	2.5 to 3.4	8.43	3.33	3.78	0.52
Accuracy (dev.) (%)	−14.0 to 1.7	0 to 5.9	2.2 to 10.2	2.3 to 3.3	−6.44	1.96	6.04	2.86
*n*	6	6	6	6	3	3	3	3

**Table 3:** Matrix effect.

Concentration (ng/ml)	TBM peak area (×10^4^)		IS peak area (×10^4^)		Ratio		Matrix effect		
	Water	M_9_	Water	M_9_	Water	M_9_	TBM	IS	Ratio
Low (120)	3.60 ± 0.12	3.83 ± 0.13	8.26 ± 0.49	8.72 ± 0.53	0.436	0.439	106	106	101
Medium (1500)	18.1 ± 1.0	20.0 ± 1.4	8.80 ± 0.77	9.30 ± 0.91	2.06	2.15	110	106	104
High (17000)	378 ± 5	413 ± 18	13.7 ± 0.5	14.7 ± 0.7	27.6	28.1	109	107	102

Data represent the mean peak area (±SD) from triplicate analysis.

**Table 4:** Stability of TBM.

Conditions		% remained	RSD (%)	*n*
In autosampler vial, 21–25°C, 3 days				
	300 ng/mL	107	3.5	3
	20000 ng/mL	105	2.4	3
In M_9_, 21–25°C, 5 days				
	300 ng/mL	104	8.1	3
	20000 ng/mL	99.2	3.6	3
3 freeze-thaw cycles				
	300 ng/mL	99.4	5.0	3
	20000 ng/mL	99.8	2.4	3
In M_9_ medium, 6 days, −70°C				
	300 ng/mL	93.8	3.3	3
	20000 ng/mL	101	3.0	3
IS (5000 ng/mL) in water		102.9	0.74	3
	24 hr, 21–25°C	74.2	3.4	3
	5 days, 21–25°C	79.5	1.7	4

**Table 5:** Interference of potential concomitant drugs.

Concentration (ng/mL)	Control	Colistin-MP^∗^	% dev.
150	150 ± 10	148 ± 8	−1.3
1500	1633 ± 61	1587 ± 98	−2.8
20000	20000 ± 557	19933 ± 737	−0.3

*Note*. Data represent the mean (SD) of triplicate analysis. ^∗^MP and colistin concentrations were 110 *µ*g/mL and 20 *µ*g/mL, respectively, corresponding to the highest concentrations in the *in vitro* model.

## Supplementary Materials

Supplementary Materials Figure S1: effect of TFA on retention time of isoniazid. Figure S2: optimization of TCA concentration.
