# Supplementary material for: Determination of Tobramycin in M9 Medium by LC-MS/MS: Signal Enhancement by Trichloroacetic Acid
Source: J Anal Methods Chem. 2018 Apr 26;2018:7965124. doi: 10.1155/2018/7965124 (PMC5944200; doi:10.1155/2018/7965124)

Figure S1. Effect of TFA on retention of isoniazid. LC condition: A= 10mM NH4FA, B=MeCN, flow rate, 0.4mL/min, Isocratic mode. Column, Hypersil Silica (50x2.1 mm, 3um, Thermo-Fisher).

```
        Sample Name: "STD5000"    Sample ID: ""    File: "0116 08 Cal.wiff"
        Peak Name: "INH"    Mass(es): "138.000/121.000 Da"
        Comment: ""    Annotation: ""
Sample Index:        25
Sample Type:       Standard
Concentration:       5000.     ng/mL
Calculated Conc:     5030.     ng/mL
Acq. Date:         1/16/2008
Acq. Time:         8:25:41 PM

Modified:             Yes
Proc. Algorithm:   Analyst Classic
Bunching Factor:      4
Noise Threshold:     10.01    cps
Area Threshold:     50.04    cps
 Num. Smooths:         2
Sep. Width:           0.20
Sep. Height:          0.01
Exp. Peak Ratio:      5.00
Exp. Adj. Ratio:      4.00
Exp. Val. Ratio:      3.00
RT Window:           30.0     sec
Expected RT:          5.02    min
Use Relative RT:      No

Int. Type:         Base To Base
Retention Time:       5.02    min
Area:            6.65e+005   counts
Height:            4.71e+004  cps
Start Time:           4.66    min
End Time:             5.63    min
```

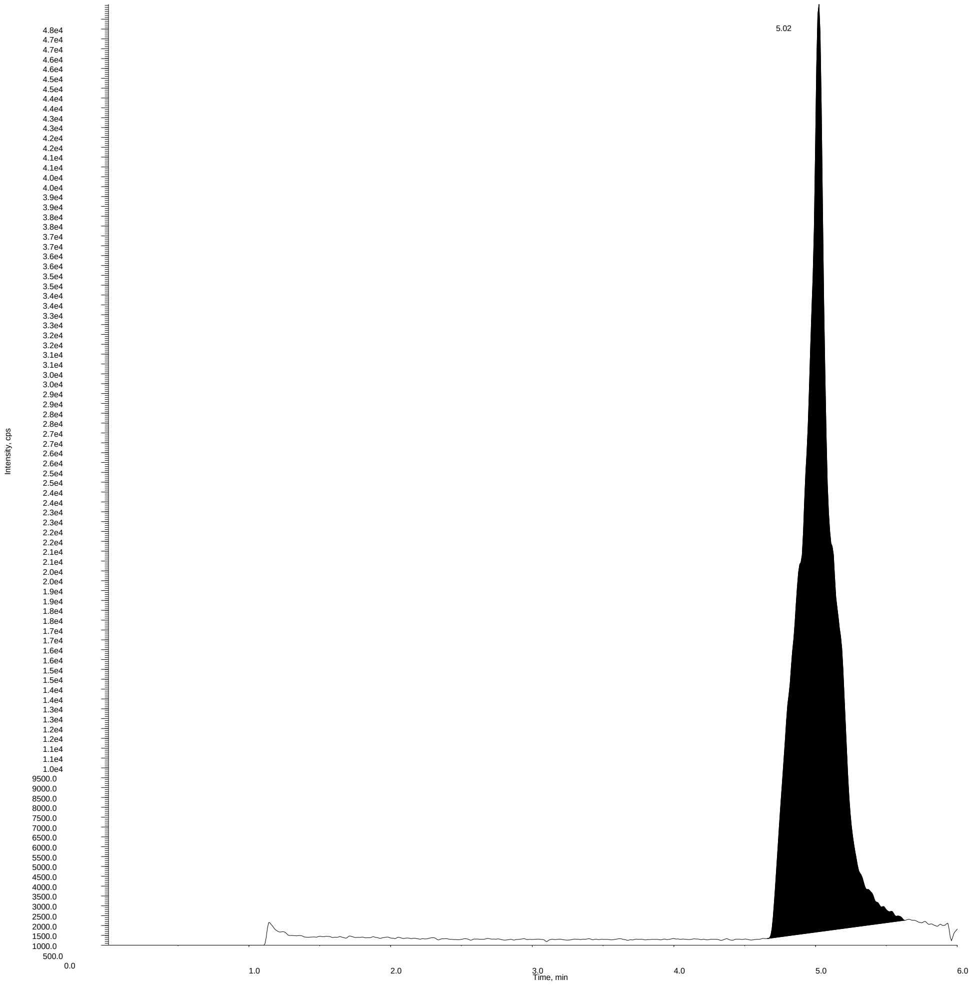


```
        Sample Name: "STD5000"    Sample ID: ""    File: "011608 noTFA.wiff"
        Peak Name: "INH"    Mass(es): "138.000/121.000 Da"
        Comment: ""    Annotation: ""
Sample Index:        30
Sample Type:       Unknown
Concentration:        N/A
Calculated Conc:   No Intercept
Acq. Date:         1/17/2008
Acq. Time:         2:37:54 AM

Modified:             Yes
Proc. Algorithm:   Analyst Classic
Bunching Factor:      4
Noise Threshold:    150.00    cps
Area Threshold:     20.00    cps
 Num. Smooths:         2
Sep. Width:           0.20
Sep. Height:          0.01
Exp. Peak Ratio:      5.00
Exp. Adj. Ratio:      4.00
Exp. Val. Ratio:      3.00
RT Window:           30.0     sec
Expected RT:          2.10    min
Use Relative RT:      No

Int. Type:         Base To Base
Retention Time:       2.10    min
Area:            4.50e+006   counts
Height:            2.39e+005  cps
Start Time:           1.67    min
End Time:             3.32    min
```

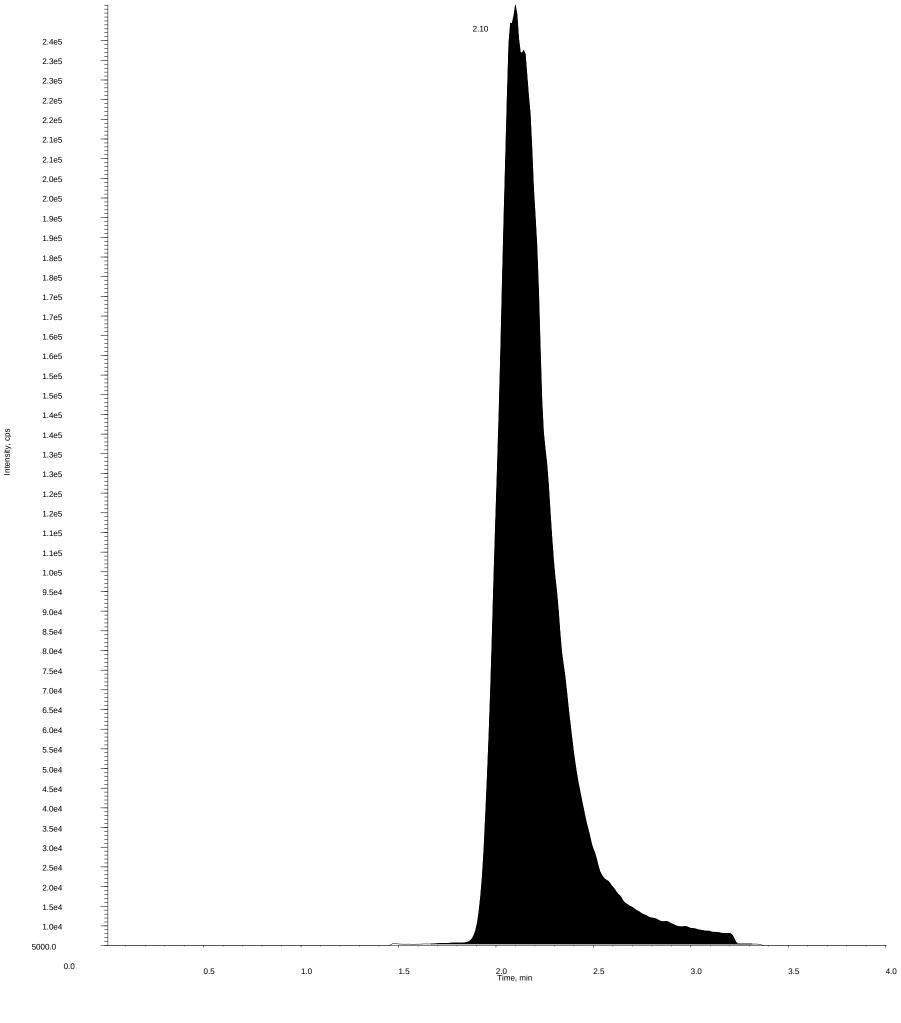

Figure S2. Optimization of TCA concentration. LC condition: A=0.14% TFA, 10mM NH4FA, B= MeCN. Flow rate, 0.6 mL/min, gradient: B% (time), 3 (0min)--3 (0.1min)--30 (2.6 min)--90(2.61min)--90(3.00min)--3(3.01min)--3(3.5min).

Sample Name: "2ugTBM_in20%TCA" Sample ID: "" File: "2012 10 22TBM_optimiz.wiff"
Peak Name: "TBM2" Mass(es): "468.100/324.000 Da"
Comment: "" Annotation: ""

Sample Index: 9
Sample Type: Unknown
Concentration: N/A
Calculated Conc: No Intercept
Acq. Date: 10/22/2012
Acq. Time: 1:41:13 PM

Modified: Yes
Proc. Algorithm: Analyst Classic
Bunching Factor: 4
Noise Threshold: 5.27 cps
Area Threshold: 26.37 cps
,Num. Smooths: 2
Sep. Width: 0.20
Sep. Height: 0.01
Exp. Peak Ratio: 5.00
Exp. Adj. Ratio: 4.00
Exp. Val. Ratio: 3.00
RT Window: 30.0 sec
Expected RT: 0.448 min
Use Relative RT: No

Int. Type: Valley
Retention Time: 0.447 min
Area: 1.18e+006 counts
Height: 5.57e+005 cps
Start Time: 0.352 min
End Time: 0.720 min

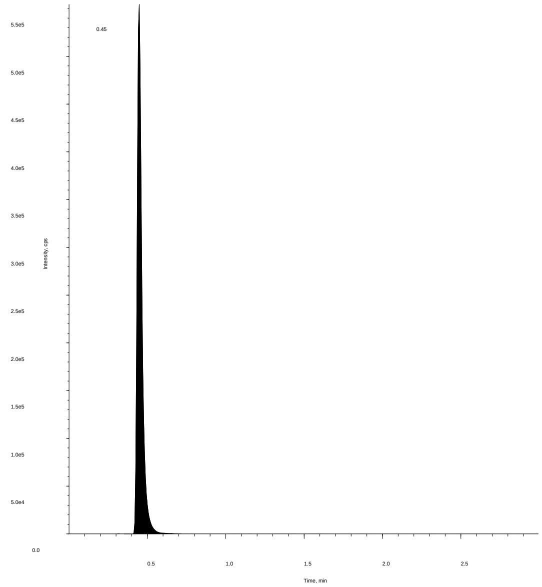

Sample Name: "2ugTBM_in2%TCA" Sample ID: "" File: "2012 10 22TBM_optimiz.wiff"
Peak Name: "TBM2" Mass(es): "468.100/324.000 Da"
Comment: "" Annotation: ""

Sample Index: 12
Sample Type: Unknown
Concentration: N/A
Calculated Conc: No Intercept
Acq. Date: 10/22/2012
Acq. Time: 1:53:01 PM

Modified: Yes
Proc. Algorithm: Analyst Classic
Bunching Factor: 4
Noise Threshold: 5.27 cps
Area Threshold: 26.37 cps
,Num. Smooths: 2
Sep. Width: 0.20
Sep. Height: 0.01
Exp. Peak Ratio: 5.00
Exp. Adj. Ratio: 4.00
Exp. Val. Ratio: 3.00
RT Window: 30.0 sec
Expected RT: 0.454 min
Use Relative RT: No

Int. Type: Valley
Retention Time: 0.456 min
Area: 1.34e+006 counts
Height: 8.41e+005 cps
Start Time: 0.403 min
End Time: 0.771 min

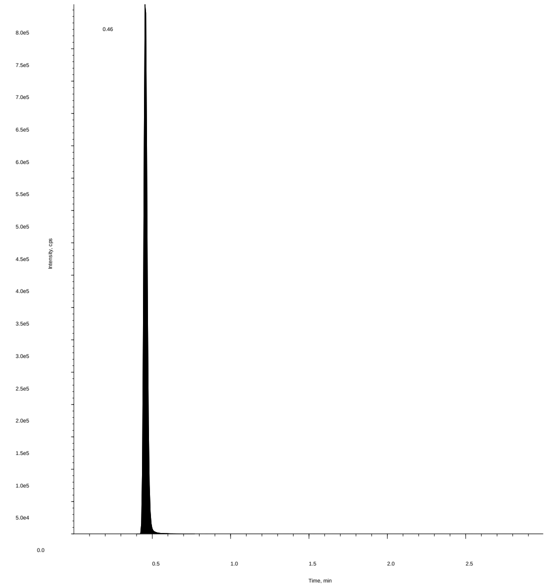

Sample Name: "1ugTBM_in10%TCA" Sample ID: "" File: "2012 10 22TBM_optimiz.wiff"
Peak Name: "TBM2" Mass(es): "468.100/324.000 Da"
Comment: "" Annotation: ""

Sample Index: 14
Sample Type: Unknown
Concentration: N/A
Calculated Conc: No Intercept
Acq. Date: 10/22/2012
Acq. Time: 2:00:55 PM

Modified: Yes
Proc. Algorithm: Analyst Classic
Bunching Factor: 4
Noise Threshold: 5.27 cps
Area Threshold: 26.37 cps
,Num. Smooths: 2
Sep. Width: 0.20
Sep. Height: 0.01
Exp. Peak Ratio: 5.00
Exp. Adj. Ratio: 4.00
Exp. Val. Ratio: 3.00
RT Window: 30.0 sec
Expected RT: 0.448 min
Use Relative RT: No

Int. Type: Valley
Retention Time: 0.448 min
Area: 5.56e+005 counts
Height: 2.94e+005 cps
Start Time: 0.403 min
End Time: 0.692 min

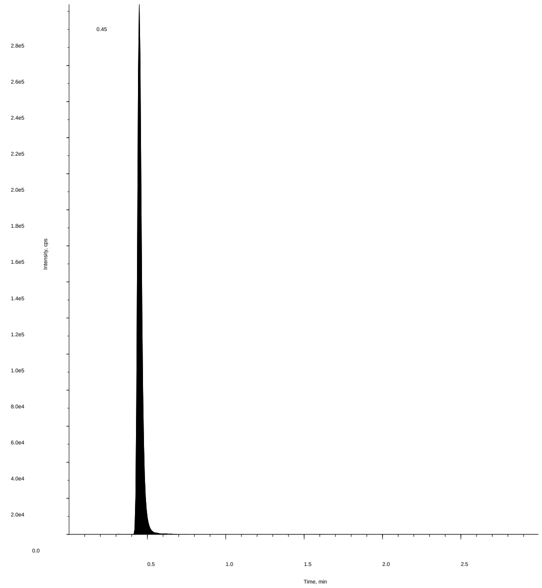

Sample Name: "1ugTBM_in1%TCA" Sample ID: "" File: "2012 10 22TBM_optimiz.wiff"
Peak Name: "TBM2" Mass(es): "468.100/324.000 Da"
Comment: "" Annotation: ""

Sample Index: 17
Sample Type: Unknown
Concentration: N/A
Calculated Conc: No Intercept
Acq. Date: 10/22/2012
Acq. Time: 2:12:43 PM

Modified: Yes
Proc. Algorithm: Analyst Classic
Bunching Factor: 4
Noise Threshold: 5.27 cps
Area Threshold: 26.37 cps
,Num. Smooths: 2
Sep. Width: 0.20
Sep. Height: 0.01
Exp. Peak Ratio: 5.00
Exp. Adj. Ratio: 4.00
Exp. Val. Ratio: 3.00
RT Window: 30.0 sec
Expected RT: 0.437 min
Use Relative RT: No

Int. Type: Valley
Retention Time: 0.437 min
Area: 5.97e+005 counts
Height: 3.77e+005 cps
Start Time: 0.391 min
End Time: 0.754 min

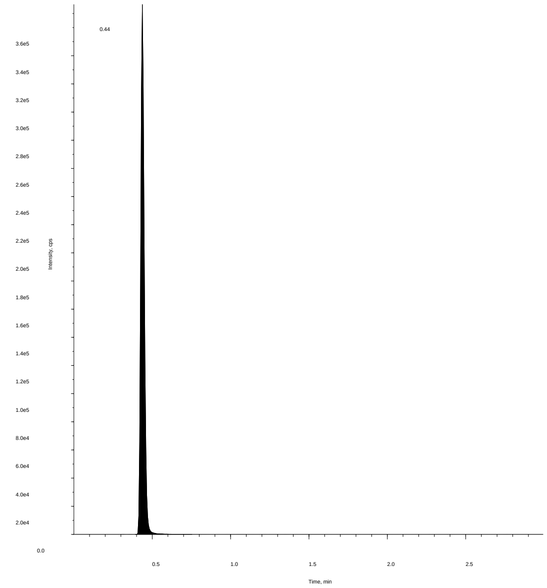

Sample Name: "50ngTBM_0.5%TCA" Sample ID: "" File: "2012 10 22TBM_optimiz.wiff"
Peak Name: "TBM2" Mass(es): "468.100/324.000 Da"
Comment: "" Annotation: ""

Sample Index: 20
Sample Type: Unknown
Concentration: N/A
Calculated Conc: No Intercept
Acq. Date: 10/22/2012
Acq. Time: 2:24:35 PM

Modified: No
Proc. Algorithm: Analyst Classic
Bunching Factor: 4
Noise Threshold: 5.27 cps
Area Threshold: 26.37 cps
,Num. Smooths: 2
Sep. Width: 0.20
Sep. Height: 0.01
Exp. Peak Ratio: 5.00
Exp. Adj. Ratio: 4.00
Exp. Val. Ratio: 3.00
RT Window: 30.0 sec
Expected RT: 0.244 min
Use Relative RT: No

Int. Type: Base To Base
Retention Time: 0.407 min
Area: 2.33e+004 counts
Height: 1.39e+004 cps
Start Time: 0.284 min
End Time: 0.584 min

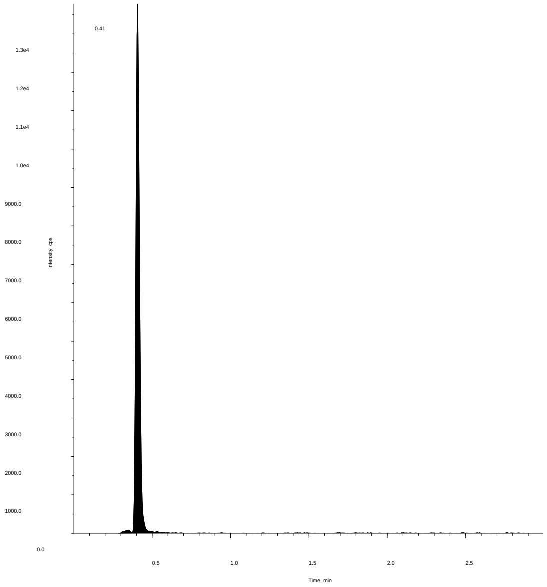

Sample Name: "2ug_inwater" Sample ID: "" File: "2012 10 22TBM_optimiz.wiff"
Peak Name: "TBM2" Mass(es): "468.100/324.000 Da"
Comment: "" Annotation: ""

Sample Index: 21
Sample Type: Unknown
Concentration: N/A
Calculated Conc: No Intercept
Acq. Date: 10/22/2012
Acq. Time: 2:29:53 PM

Modified: No
Proc. Algorithm: Analyst Classic
Bunching Factor: 4
Noise Threshold: 5.27 cps
Area Threshold: 26.37 cps
,Num. Smooths: 2
Sep. Width: 0.20
Sep. Height: 0.01
Exp. Peak Ratio: 5.00
Exp. Adj. Ratio: 4.00
Exp. Val. Ratio: 3.00
RT Window: 30.0 sec
Expected RT: 0.244 min
Use Relative RT: No

Int. Type: Base To Base
Retention Time: 0.333 min
Area: 5.63e+004 counts
Height: 2.23e+004 cps
Start Time: 0.289 min
End Time: 0.681 min

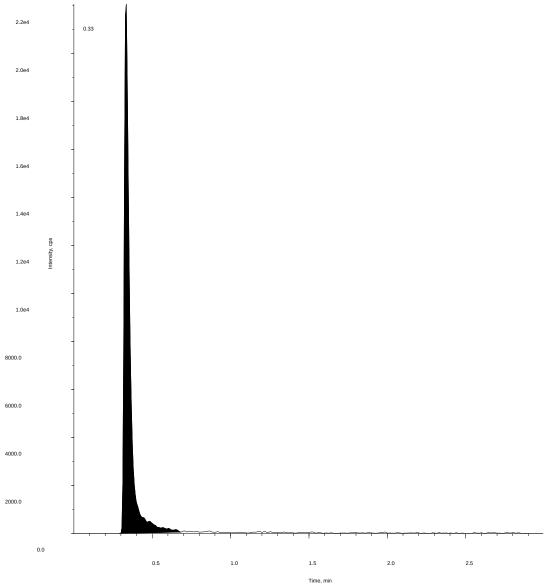

Supplement: Supplementary Materials — Figure S1: effect of TFA on retention time of isoniazid. Figure S2: optimization of TCA concentration. [file 7965124.f1.pdf]
